# Shortening spinal column reconstruction through posterior only approach for the treatment of unstable osteoporotic burst lumber fracture: a case report

**DOI:** 10.1007/s00402-012-1653-x

**Published:** 2012-11-20

**Authors:** Ahmed Shawky, Markus Kroeber

**Affiliations:** Center for Spine Surgery, Hirslanden Clinic St. Anne, St. Anne Strasse, 32, 6003 Lucerne, Switzerland

**Keywords:** Osteoporotic fracture, Posterior corpectomy, Shortening reconstruction, Unstable burst fracture, Thoracolumbar fracture

## Abstract

**Study design:**

Case report.

**Clinical question:**

This study reports if shortening reconstruction procedure through posterior approach only can be used in osteoporotic unstable fracture as well as post-traumatic burst fracture.

**Methods:**

An 80-year-old female patient with unstable burst osteoporotic fracture of L1 underwent posterior approach corpectomy and shortening reconstruction of the spinal column by non-expandable cages.

**Result:**

The surgery was uneventful, with average blood loss. Using of small profile cages has helped us to avoid root injury. Augmentation of the screw with cement and the compressive force applied to the spine column aids in obtaining a rigid construct with good alignment without any neurological complication.

**Conclusion:**

Shortening reconstruction procedure through only posterior approach is a viable option in treating unstable osteoporotic fracture as well as post-traumatic fractures. Using non-expandable cage is advocated to avoid cage subsidence.

## Introduction

Osteoporotic compression fractures are a leading cause of disability and mortality in the elderly [1]. Vertebral fracture contributes to pain and disability and is associated with decline in physical performance even when the pain is not reported. Indeed, the adverse effect of vertebral fracture on most activities of daily living is almost as great as that seen in hip fracture [2].

If a complex osteoporotic fracture is present which means a concomitant neurological compression and/or a severe spinal deformity, open surgical treatment is advocated. In this case, a combination of cement reinforcement and internal fixation might be necessary to achieve sufficient stability [3].

One option for the treatment of unstable thoracolumbar burst fractures is a single posterior corpectomy and reconstruction using various recently introduced cages [4–6]. We present this case to report that this approach can be used also in unstable thoracolumbar osteoporotic fracture as well as post-traumatic types.

## Case report

### Clinical presentation

An 80-year-old female patient presented to our hospital complaining of progressive back pain not responding to conservative treatment which disabled her from walking. She has a recent history of post-traumatic osteoporotic compression fracture L1 type A1-1 according to AO Classification. This was diagnosed and treated conservatively at another institution 4 months back.

#### Evaluation and management

**Fig. 1:**
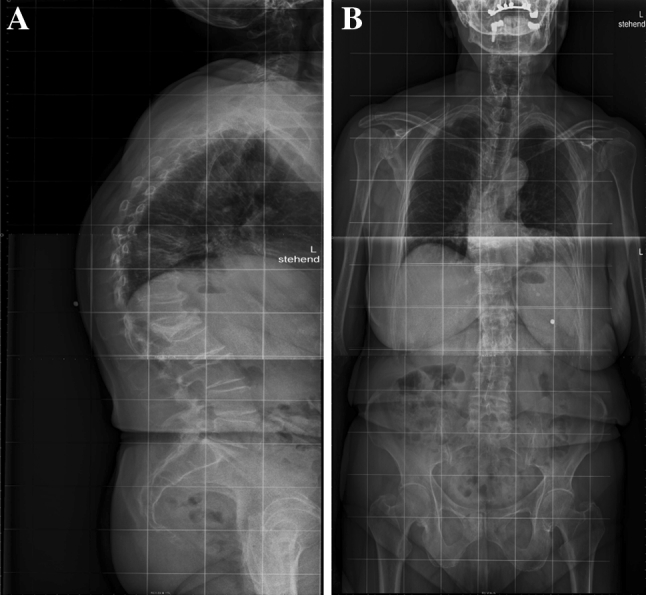
Showing the fractured L1 body with sever kyphosis. Also the old consolidated T9 fracture that produce more kyphotic deformity

**Fig. 2:**
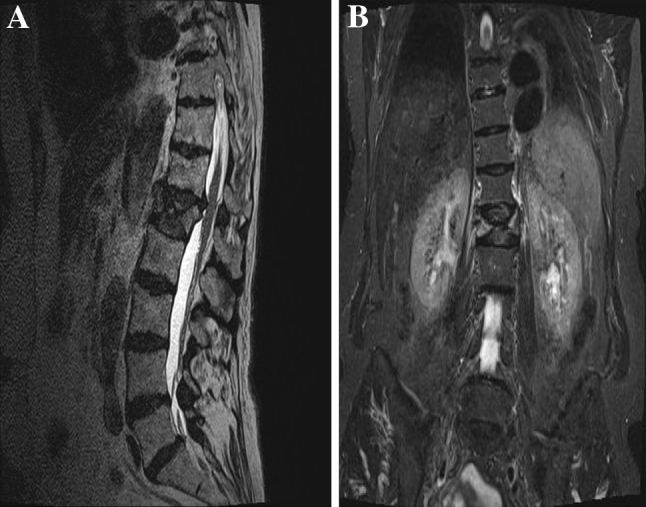
MRI showing the damaged both endplates with the retropulsed fragment

The patient was evaluated both clinically and radiologically by X-ray and MRI. She was found to have non-consolidated burst fracture L1 type A3-2 with hyperkyphotic angle 30° (measured between the superior endplate of the level above and the inferior one of the level below), beside an old consolidated compression fracture T9 (Fig. 1). MRI showing damaged both superior and inferior endplate with severe instability and retropulsed fragment into the spinal canal (Fig. 2).

### Surgical technique

Patient was placed prone, and posterior midline incision is made. Pedicular screws augmented with cement are inserted into two levels above and below the fractured L1. The lamina and the bilateral articular processes of the affected segment are resected carefully; lower half of the lamina above and upper half of the lamina below are resected too.

Both pedicels are removed, then the fractured vertebral body is drilled and resected by a rongeur. The cranial and caudal discs are removed including the cartilaginous end plates. Rods are applied to the screws, two un-expandable cages filled with milled bone are inserted followed by compressive force to correct the angular deformity, shortening the spinal column and fix the cages. All of these steps were done under neuromonitoring. The remaining milled bone is embedded on the postero-lateral side for facet fusion.

**Fig. 3:**
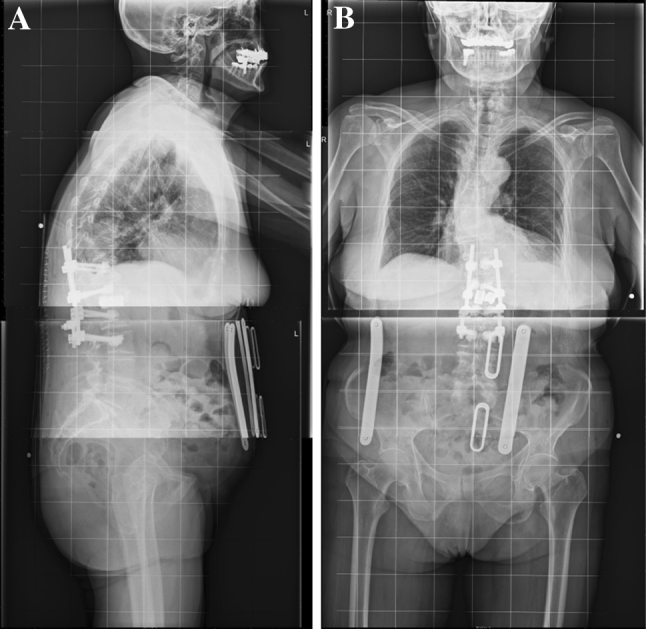
Post operative X ray showing good alignment and stable implants

Post-operatively patient was vitally stable, fully neurologically intact with controllable pain at the surgical site. She was fitted with a thoracolumbar orthosis. The post-operative X-ray shows good stable alignment spine with nice cage position, the kyphotic angle between T12 and L2 increased to 170° (Fig. 3).

## Discussion

In older women with vertebral fracture, hyperkyphosis predicts an increasable risk for death, independent of underlying spinal osteoporosis [7]. Thoracolumbar fracture, mid portion fracture type and involvement of vertebral posterior wall are risk factors for progressive collapse following acute osteoporotic spinal fracture [8].

Our objectives for this case were to correct the angular deformity and stabilize the spinal columns through single surgical approach to avoid the disadvantages of anterior approach in this old patient. Anterior decompression and shortening reconstruction with a titanium mesh cage using only a posterior approach offer several advantages over traditional anterior or combined anterior–posterior approach for the unstable lumber burst fractures. However, further modification is required for the procedure to prevent the subsidence of the cages, especially in patients with low BMD either by inserting a cage larger than 22 mm which seems to be difficult without injuring the nerve roots, or usage of multiple cages may be a solution [9]. During our surgery, it was so easy to insert two un-expandable cages from right and left with minimal root retraction. After compression, we checked the cages position by applying some force on them and they were stable enough to support the spinal column

Haiyan et al. [5] reported a similar three-column reconstruction of thoracolumbar fractures above L2 through single posterior approach. They noted that use of an adequate shorter non-expandable cage can provide sufficient biomechanical stability. Shortening reconstruction for unstable burst fracture with a shorter cage appears to offer several advantages; acute spinal column shortening within safe range increases the spinal cord blood flow which is important for cord function recovery [10]. Biomechanical compression among different cages has no significant difference [11]. Resected local bone maintains bone grafting without donor site morbidity.

## Conclusion

The current case demonstrates that the shortening spinal column reconstruction procedure through only posterior approach can be used safely for the unstable osteoporotic fractures as well as post-traumatic burst features type to avoid the complication of anterior approach in such fragile patient. To avoid the cage subsidence in such fragile bone, the usage of one or more non-expandable cage is advocated.
